# Hydrophobicity Tuning by the Fast Evolution of Mold Temperature during Injection Molding

**DOI:** 10.3390/polym10030322

**Published:** 2018-03-15

**Authors:** Sara Liparoti, Roberto Pantani, Andrea Sorrentino, Vito Speranza, Giuseppe Titomanlio

**Affiliations:** 1 Department of Industrial Engineering, University of Salerno, via G. Paolo II 132, 84084 Fisciano (SA), Italy; sliparoti@unisa.it (S.L.); rpantani@unisa.it (R.P.); gtitomanio@unisa.it (G.T.); 2 Institute for Polymers, Composites and Biomaterials (IPCB-CNR), via Previati n. 1/E, 23900 Lecco, Italy; andrea.sorrentino@cnr.it

**Keywords:** hydrophobicity, contact angle, polypropylene, atomic force microscopy, injection molding, mold temperature evolution

## Abstract

The surface topography of a molded part strongly affects its functional properties, such as hydrophobicity, cleaning capabilities, adhesion, biological defense and frictional resistance. In this paper, the possibility to tune and increase the hydrophobicity of a molded polymeric part was explored. An isotactic polypropylene was injection molded with fast cavity surface temperature evolutions, obtained adopting a specifically designed heating system layered below the cavity surface. The surface topology was characterized by atomic force microscopy (AFM) and, concerning of hydrophobicity, by measuring the water static contact angle. Results show that the hydrophobicity increases with both the temperature level and the time the cavity surface temperature was kept high. In particular, the contact angle of the molded sample was found to increase from 90°, with conventional molding conditions, up to 113° with 160 °C of cavity surface temperature kept for 18 s. This increase was found to be due to the presence of sub-micro and nano-structures characterized by high values of spatial frequencies which could be more accurately replicated by adopting high heating temperatures and times. The surface topography and the hydrophobicity resulted therefore tunable by selecting appropriate injection molding conditions.

## 1. Introduction

Natural hydrophobicity and superhydrophobicity is grabbing the interest of many researchers and has inspired mimetic attempts in recent years [1,2,3]. The surfaces are characterized by high contact angle show self-cleaning [4,5], water repellence capabilities [6] and corrosion inhibition [7], and thus, thus they are very interesting for biomedical and pharmaceutical fields too [8,9,10].

Artificial super-hydrophobicity are obtained through the hierarchical structuring of a surface, namely by the formation of both micro and nano-structures on the surface [11,12,13]. The technologies adopted for the production of micro and nano-structured surfaces are classified as bottom-up or top-down methods. The bottom-up techniques take advantages of the self-assembling capabilities of the molecule thus the formation of a structured surface takes place layer by layer [14]. These methods can be adopted for a limited range of materials and are not suitable for mass production [15]. The top-down approaches consist in etching away bulk material to achieve the required smaller structural architectures [16], or in replicating master geometries [17]. These methods can be applied to a wide range of material, and are suitable for mass production of micro and nano-structured surfaces [15,18]. In particular, the “replica molding” techniques consist in the replication of a master, produced by lithography-based techniques, by hot embossing or injection molding [16,19]. The latter surely presents the advantage of cheaper and faster production [20,21,22,23,24]. Yamaguchi et al. [25] proposed to replicate a master having nano-periodic structures, obtained by a femtosecond laser, layered on the cavity surface. They studied the effect of nano-structures replica on the wettability of acrylonitrile-ethylene-styrene moldings and found that the apparent contact angle increases with respect to the surface obtained on a smoother cavity. Puukilainen et al. [26] adopted a cavity with micro-pillars in micro-injection molding of polypropylene. They found that the distance between the pillars is the key parameter in tuning the surface wettability. Yoo et al. [27] adopted injection molding process to replicate nano-structured masters, having nano-pillars, on polypropylene and polycarbonate objects. They found that the replication accuracy increases with the mold temperature and, more important, that the hydrophobicity also increases with the mold temperature. The same research group [28] obtained similar results on polyethylene copolymer. All these studies highlight that the topography of a surface and, in particular, the quality of the replication of micro and nano-structures, directly influences the wettability of a surface. On their turn, all the processing parameters that control the accuracy of the replication influence also the wettability. Thus, it is possible to tune the wettability by controlling the same processing conditions that influence the replication accuracy. However, the replication of micro and nano-structures by injection molding process is not an easy task. The formation of a frozen layer on the polymeric surface, due to the contact of the molten polymer with the cold cavity surface, prevents an accurate replication [29]. It is necessary to increase the mold temperature, but this induces an increase of the processing time. The methods adopted to obtain an evolution of the mold temperature require additional tool cost and cause a significant increase in processing time [23,30,31,32,33,34].

In this paper, a fast evolution of mold temperature was obtained adopting a thin heating device, made of carbon black loaded poly(amide-imide), that is able to remarkably increase the temperature of a selected area on the cavity surface in few seconds, and to control this temperature level for a given time. This innovative heating device, as a result of its reduced thickness, 50 μm, also allows for a fast decrease in temperature soon after its deactivation. The fast evolution of the cavity surface temperature allows for a shorter processing time. The injection molding process coupled with such a heating system was adopted to obtain a better replication of a commercial steel layer. This paper aims to demonstrate that the control of the replication of a random structured surface by injection molding allows for tuning the hydrophobicity of polypropylene surfaces.

## 2. Materials and Methods

An isotactic polypropylene (iPP, Basell T30G, Ferrara, Italy), having an average molecular weight of *M*_w_ of 376,000 g·mol^−1^, a polydispersity index *M*_w_/*M*_n_ of 6.7, and a meso pentads content of 87.6% was adopted. A complete mechanical, rheological and thermal characterization of this polymer can be found elsewhere [35,36].

Injection molding tests were carried out by adopting a melt temperature of 220 °C, a mold temperature of 28 °C and a holding pressure of 26 MPa. Table 1 summarizes all the other operating conditions.

**Figure 1:**
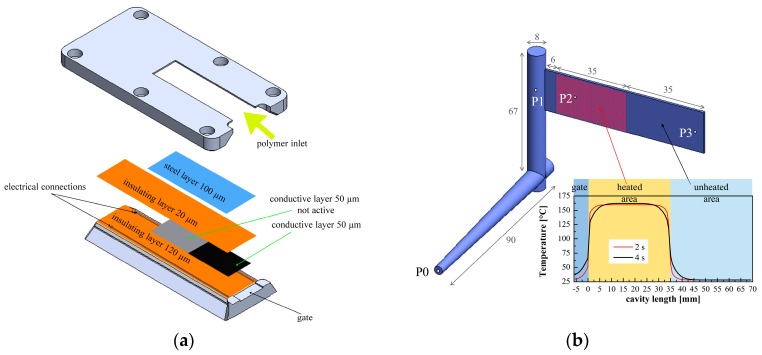
(**a**) Schematic drawing of the mold insert adopted to layer the heater below the cavity surface; (**b**) Sketch of the cavity adopted for the injection molding experiments (measures are in mm), pressure transducer positions (P0–P3) are indicated. A sketch of the surface temperature profile along the flow path is also reported.

A rectangular cavity having a length of 70 mm, a width of 20 mm, and a thickness of 1 mm was adopted. A thin heater device, made of poly(amide-imide) loaded carbon black, was layered under the cavity surface. The heater was electrically insulated from the mold by poly(amide-imide) layers, having a thickness of 0.020 mm. An additional insulating layer 0.120 mm thickness was located between the heater and the mold to reduce the heat loss. A protective steel layer (0.100 mm thickness) covering all the length of the cavity was adopted to avoid the direct contact of the melt with the heating device. Supplementary information about the heating device is reported elsewhere [37]. The heating device adopted allows for heating a selected area on the cavity surface. Figure 1 shows a sketch of the heating device layered in the mold insert and a sketch of the cavity with the pressure transducer positions. In the configuration adopted in this work and depicted in Figure 1a, the cavity surface was only partially heated (for the first 35 mm adjacent to the gate).

As a reference, injection molding experiments were carried out in conventional injection molding conditions (CIM). In these cases, the temperature of the cavity surface at the beginning of the experiment corresponds to the temperature of the whole mold (28 °C). All the other experiments were performed adopting the heating device to increase the temperature of the cavity surface (see Figure 1b). The activation of the heating device on the half cavity length allows for comparing the contact angle of a surface obtained with high cavity temperature with the contact angle of a surface obtained with low cavity surface temperature, directly on the same sample. The heating devices were automatically activated with constant electrical power 0.5 s after the start of the injection, which corresponds to about 2 s before the contact with the polymer in position P2. The temperature reached on the cavity surface, which depends on the adopted electrical power, after 10 s heating time is denoted as *T*_level_ in the Table 1. 80, 120 and 160 °C were selected as *T*_level_. Different heating times, 0.7 s (corresponding to the cavity filling time), and 18 s were adopted. In the following, each test was coded by sequencing the cavity surface temperature and the heating time (i.e., 160-18 refers to an experiment obtained with 160 °C cavity surface temperature and 18 s heating time).

Figure 1b also shows a sketch of the temperature profile along the flow path. It is possible to observe that the boundaries of the heated area are characterized by a temperature that is below the selected temperature, 160 °C in this case.

The surface of the injection molded samples was observed by Atomic Force Microscope (AFM). The area, 60 × 60 µm^2^, for the AFM acquisitions was selected in order to include all the representative structures. Micro-graphs in air at room temperature were collected with a Dimension 3100 coupled with a Bruker Nanoscope V controller (Bruker, Billerica, MA, USA ), operating in tapping mode. For each position along the flow direction, a square area of 3600 μm^2^ was investigated with a resolution of 512 points/line and a scan rate of 0.5 line/s. Commercial probe tips (mod. OTESPA, Bruker, Billerica, MA, USA) with nominal spring constants of 42 N/m, resonance frequency of 300 kHz, tip with radius of 7 nm and height of 7–15 μm were adopted. AFM topographic maps were acquired with NanoScope software version 7.30 (Bruker, Billerica, MA, USA). To remove tilt or bow from the acquired surface a flatten procedure was applied by the NanoScope Analysis software version 1.80 (Bruker, Billerica, MA, USA); to remove noise a low pass filter with a cutoff frequency of 2 μm^−1^ was applied. The NanoScope Analysis software also allowed for performing the roughness and the frequency analysis of the acquired patterns.

The processed AFM acquisitions were analyzed by the software MountainsMap 7.1 (by DigitalSurf, Besançon, France) to obtain the 3D topographic parameters *S*_da_ (average close dale area), *S*_ha_ (average close hill area) and *S*_dr_ (developed area ratio) according to the standards defined by EUR15178N and ISO25178. The dale area, *S*_da_, and hill area, *S*_ha_, are calculated as the mean area of all individual motifs used to segment the surface acquired by AFM. The considered areas are the horizontal areas of the motifs projected onto the horizontal plane [38]. The developed area ratio *S*_dr_ is calculated by summing the local areas when following the surface curvature and it represents the area excess with respect to the projected area. It is expressed as the percentage of the projected area [38].

Contact angle measurement is an accurate method for determining the interaction energy between a liquid and a solid. Static contact angle measurements were carried out at room temperature with distilled water. A water drop (5 μL) was placed on the sample and photographed once a second for 30 s. The contact angle was determined mathematically by fitting a Young-Laplace curve around the drop. Values recorded between 6 and 30 s were averaged to obtain the contact angle for each measurement. The measurements were repeated on a series of three equivalent samples and mediated to calculate the contact angle value on each point of the molded sample. The contact angles were measured along the flow path.

## 3. Results

### 3.1. The Process

**Figure 2:**
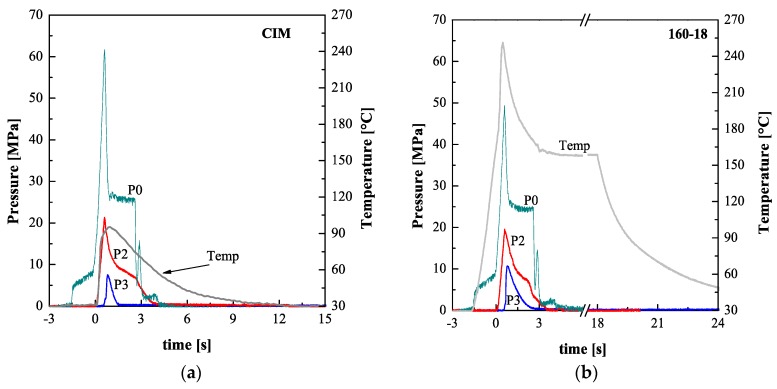
Pressure and temperature evolutions measured during the injection molding experiments conventional injection molding (CIM) (**a**) and 160-18 (**b**).

Figure 2 shows, for two representative experiments, the pressure in three positions inside the cavity, along the flow path, P0, P2 and P3 (see Figure 1), and the temperature evolutions measured in position P2. Figure 2a shows pressure and temperature evolutions recorded during CIM, the experiment performed without the heating device. Figure 2b shows the pressure and the temperature evolutions recorded during the experiment named 160-18.

The pressure in position P0 was recorded in the injection chamber and represents the pressure imposed on the melt by the machine; it reached a maximum at the end of the filling stage (*t* = 0.7 s). This pressure is affected by the cavity surface temperature: as the cavity surface temperature increases the pressure necessary to fill the cavity at the set flow rate decreases. After the pressure peak, for both cases reported in Figure 2, the pressure in position P0 reaches the value set as holding pressure, 26 MPa, and is kept constant for 2 s, after that it decreases down to zero. When the polymer comes in contact with the cavity walls (*t* = 0 s), the pressure in position P2 starts to increase. It reaches a maximum value of about 20 MPa at the end of the cavity filling. The final filling pressure in position P2, at *t* = 0.7 s, is also affected by the adopted cavity surface temperature: it decreases as the cavity surface temperature increases. After the filling end, the pressure in position P2 decreases down in two steps: during the first step, the density increase (induced by the polymer cooling and crystallization) is counteracted by the packing flow; during the second step, the pressure decrease is faster due to the pressure release in position P0 [39]. When the melt contacts the cavity in position P3, the filling is almost complete; thus, the pressure evolution in this position is mainly driven by the packing flow. The pressure drop, determined by the packing flow, and the polymer viscosity are both smaller with higher cavity temperature, thus, the pressure in position P3 is higher during the experiment 160-18. The pressure decay in position P3, is faster with respect to the pressure decay in positions P2, for both cases shown in Figure 2, because the packing flow does not efficiently counteract the cooling. Figure 2 also shows the temperature evolutions in position P2. When the heating device is not present (Figure 2a) the temperature reaches 90 °C at the first contact of the hot melt with the cavity wall, afterward, the temperature decreases due to the contact with the cold cavity walls. Figure 2b shows that, at the contact with the melt, the surface temperature, which had already raised because device heater activation, undergoes an additional immediate increase and, soon after (see Figure 2b), a fast decrease toward a constant value denoted as *T*_level_. At the heater deactivation, the temperature decreases down to the value of the whole mold (28 °C).

### 3.2. Contact Angle Distribution

**Figure 3:**
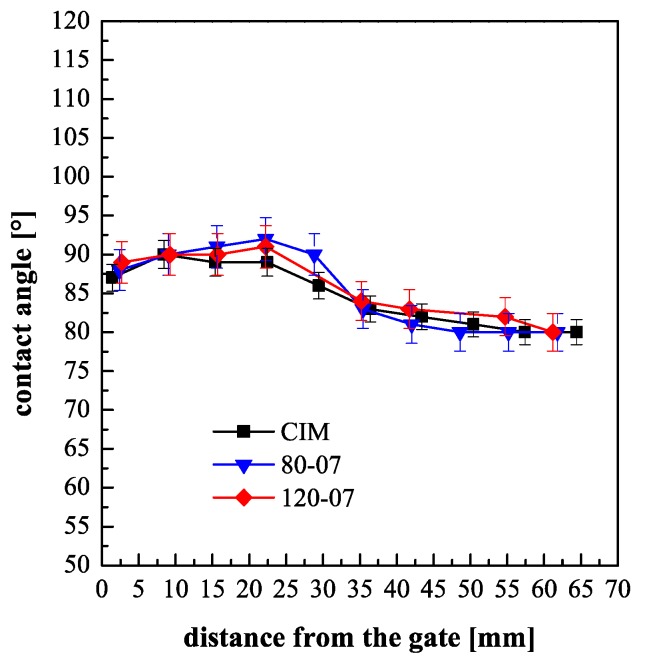
Contact angles of the molded samples obtained with CIM and different cavity surface temperature, keeping active the heating device only during the filling.

In the literature, it has been demonstrated that the cavity temperature influences the hydrophobicity of the molded objects [27]. This paper aims to analyze the effect of the temperature evolution, namely the cavity surface temperature and the heating time, on the molded part surface hydrophobicity. Figure 3 shows the contact angle distributions along the flow path of the samples obtained with different cavity surface temperatures and 0.7 s heating time. The contact angle distribution for the experiment named CIM is also reported for comparison.

For all the considered samples, the contact angles show a similar distribution. When a heating time equal to the filling time, 0.7 s in these cases, was adopted, the contact angles showed values that have a negligible dependence on the temperature set on the cavity surface, within the measurement reproducibility. In particular, the contact angle distributions of the samples obtained with different cavity surface temperatures are similar to the one obtained for the CIM sample. These values show a slight decrease on increasing the distance from the gate, in agreement with the fact that both pressures and temperatures decrease on increasing the distance from the gate.

**Figure 4:**
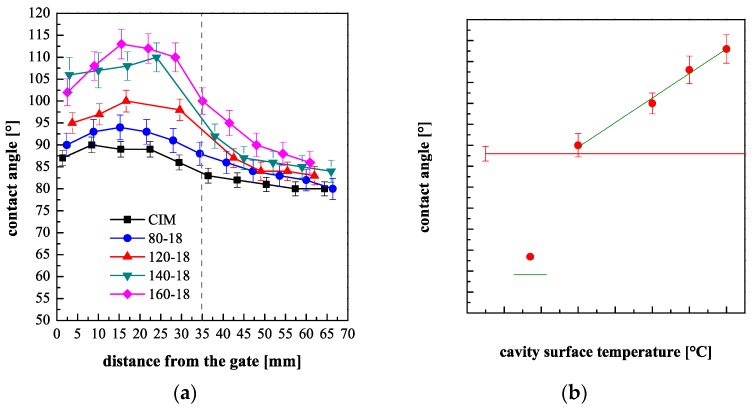
(**a**) Contact angles of the molded samples obtained with CIM and different *T*_level_, keeping active the heating devices for 18 s. The vertical dashed line identifies the location of the heating element; (**b**) Contact angle dependence on the cavity surface temperature (the contact angles were measured at 20 mm distance from the gate on the samples obtained with 18 s heating time).

Figure 4a shows the contact angle distributions along the flow path, measured for the samples obtained with different cavity surface temperatures and 18 s heating time. The contact angle distribution along the flow path for the sample CIM is also reported for comparison. Figure 4b shows the plot of contact angles measured at 20 mm distance from the gate (namely at the center of the heated area) vs. the *T*_level_.

In these cases, since the heating device is kept active for long times, the cavity surface temperature has a strong influence on the contact angle: the contact angles show higher values, with respect to the CIM sample, especially at the central part of the heated area. The distributions of the contact angle present a maximum, for all the considered cases, because, as shown in Figure 1b, the temperature in the first 10 mm downstream the gate is smaller than *T*_level_, due to the contact with a cold gate. On plotting the contact angle measured at 20 mm downstream the gate versus the *T*_level_, a linear dependence is obtained, as shown in Figure 4b, in the whole range of set temperature explored (80–160 °C).

It can be concluded that the increase in the cavity surface temperature up to 160 °C, allows for a contact angle increase up to 28% with respect to the contact angle measured for the CIM sample. Expensive masters with specially designed structures were proposed in the literature to obtain a similar increase of the contact angle by injection molding process. In this work, this result was achieved adopting a commercial and cheap steel layer and by an evolution of the cavity surface temperature.

### 3.3. Analysis of the Topography of the Molded Surface

**Figure 5:**
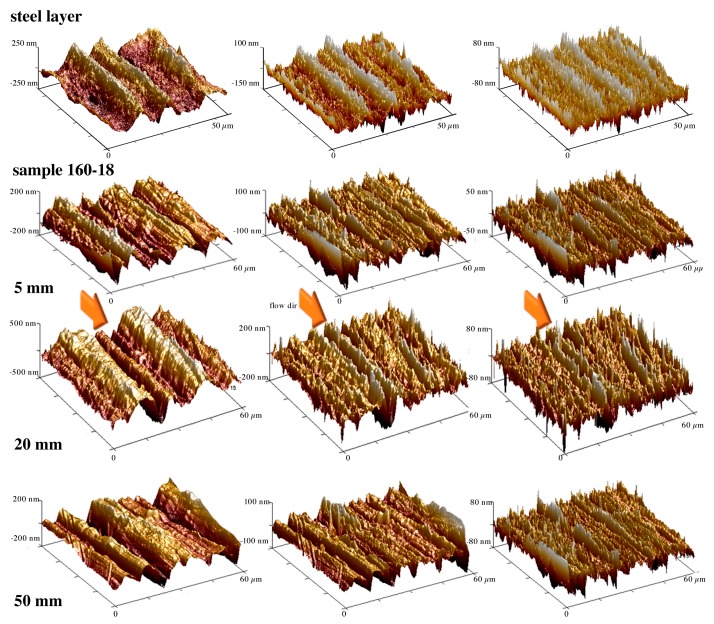
Topographies of the steel layer and of the sample 160-18 at different distances from the gate. From left to right are reported the atomic force microscopy (AFM) maps processed by applying a flatten procedure and two high pass horizontal filters, 0.1 and 0.2 µm^−1^ cut off frequency.

In the literature, many authors correlated the hydrophobicity of a surface to its topography [25,40]. The presence of a hierarchical structure on a surface induces an increase of the hydrophobicity with respect to a smooth surface [41]. This was assessed by replicating masters with complex surfaces on polymeric surfaces. In this work, a commercial steel layer is adopted as a master. The AFM acquisitions, shown in the uppermost of Figure 5, confirm that the steel layer is characterized by a complex surface, being composed of both micro and nano-structures, tightly packed. In particular, the components on different scales were highlighted applying high pass filters to the flattened maps, with cut off frequencies of 0.1 and 0.2 µm^−1^. These frequencies attenuate by 50% and 70%, respectively, the spectral component of the signal acquired on the steel layer.

Since the steel layer is composed of structures on different scales, the hydrophobicity is expected to increase by the accurately replicate the steel layer surface on the molded object. Figure 5 shows the topographies acquired on the sample 160-18 at different positions, 5, 20 and 50 mm, downstream the gate, where differences in contact angles were detected.

Figure 5 shows that also the surfaces of the 160-18 samples are characterized by sub-micro and nano-structures in all the positions along the flow path. To compare the structures and determine the accuracy of the replication, a roughness analysis was carried out and summarized in Table 2.

Table 2 shows that the roughness of the molded samples is nearly the same for both CIM and 160-18 samples and that the differences in the roughness values along the flow path are within the reproducibility of the measurements. Thus, the roughness is not sufficient in describing the replication accuracy of the surfaces. A deeper analysis of the profiles is necessary.

**Figure 6:**
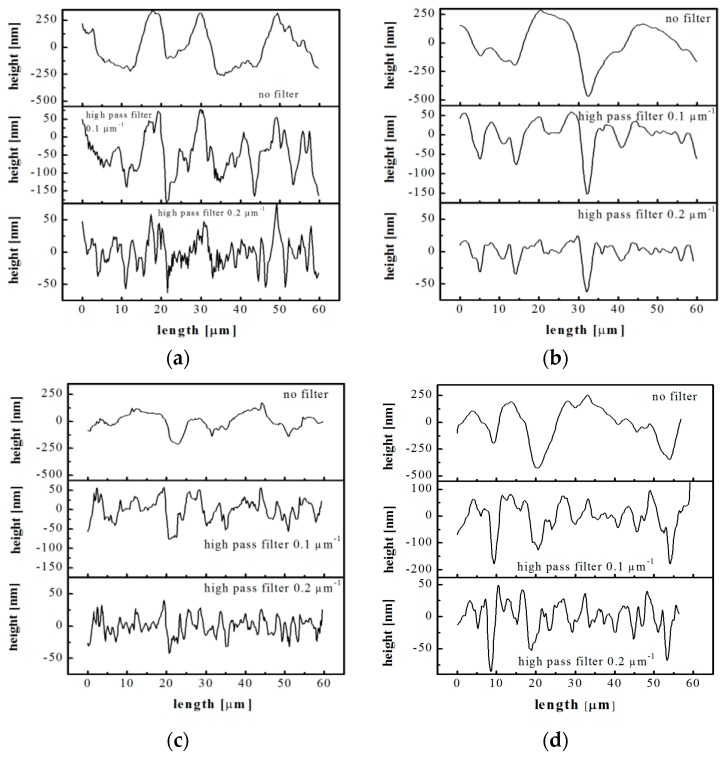
AFM patterns obtained from the maps acquired on the (**a**) steel layer; (**b**) CIM sample (20 mm distance from the gate); (**c**) 160-18 sample (20 mm distance from the gate) and (**d**) 160-18 sample (50 mm distance from the gate). Three patterns are reported for each sample: the flattened pattern, and the patterns obtained applying 0.1 and 0.2 µm^−1^ high pass filters.

Figure 6 shows some profiles, obtained by the AFM maps acquired on the samples CIM and 160-18 (20 and 50 mm distance from the gate). The profile of the steel layer is also reported for comparison. Three patterns are reported for each sample: the profile obtained from the flattened maps and two additional profiles obtained applying high pass filters with cutoff frequencies of 0.1 and 0.2 µm^−1^ to the AFM maps.

**Figure 7:**
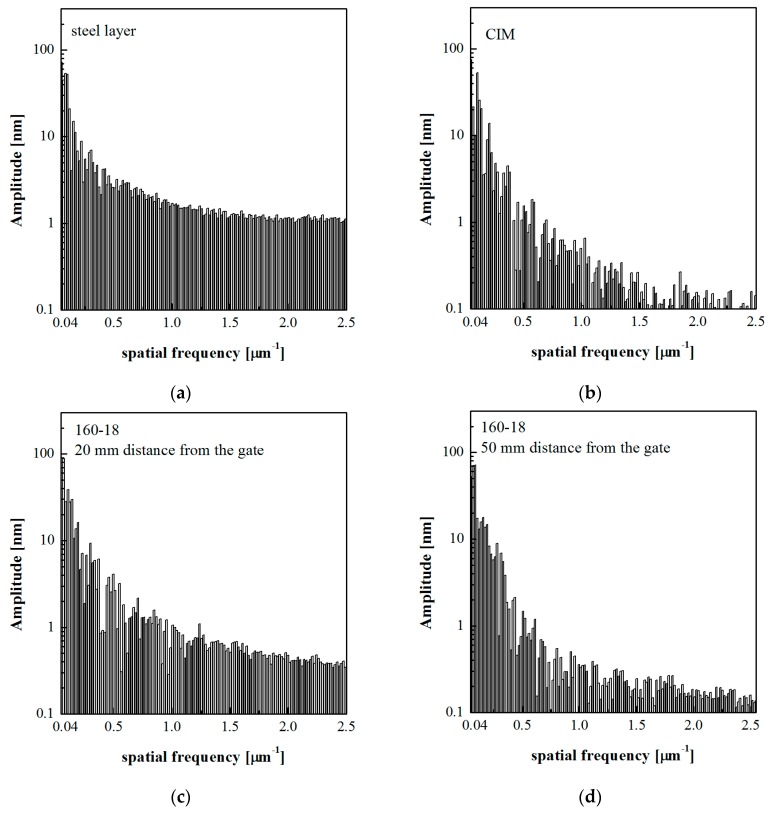
Spatial frequency analysis of the AFM acquired signal for (**a**) the steel layer and for the molded samples (**b**) CIM (20 mm distance from the gate), (**c**) 160-18 (20 mm distance from the gate) and (**d**) 160-18 (50 mm distance from the gate).

Figure 6a, related to the steel layer, shows that the profile obtained from the flattened map is composed of structures on micro and nanoscale. Figure 6b shows that the pattern of CIM sample is smoother than the corresponding pattern of the steel layer and also with respect to the 160-18 sample obtained in the heated area (Figure 6c). This characteristic of the CIM sample is evident especially if comparing the patterns obtained after the application of 0.2 µm^−1^ high pass filter. The patterns of the sample 160-18 acquired at 50 mm distance from the gate (Figure 6d), appear smoother than the patterns acquired on the same sample but in the heated area (20 mm distance from the gate, Figure 6c). The mold temperature increase induces a better replication of the structures that are present on the cavity surface, in both micro and nano-metric scales [23,24,42]. This is the case of the 160-18 sample at 20 mm distance from the gate. Frequency analyses were performed to quantify the replication accuracy of the random surface of the steel layer. Figure 7 shows the harmonic content related to the flattened patterns of the steel layer and of the samples CIM and 160-18, the latter at two distances from the gate, 20 mm and 50 mm.

The frequency analysis of the AFM patterns shows that the steel layer is composed by significant contributes with high frequencies, up to 2.5 µm^−1^, which correspond to structures of smaller dimensions. The CIM sample is characterized by a harmonic content completely different with respect to the steel layer, with significant contributes at the lower frequencies, and negligible contributes at the higher frequencies. The acquisition carried out at 20 mm distance from the gate on the sample 160-18 is characterized by a harmonic content similar to that one observed for the steel layer. The acquisition of the 160-18 sample at 50 mm distance from the gate shows a behavior similar to the CIM sample. This is consistent with a poor replication accuracy and also with the lower values of the contact angles. The frequency analysis performed on the samples considered in this paper show that the behavior became more similar to the one depicted in Figure 7c increasing *T*_level_. Thus, on the basis of the frequency analysis, it is possible to conclude that the 160-18 sample, at 20 mm distance from the gate, replicates more accurately the steel layer. Being this latter characterized by a considerable portion of structures having high spatial frequencies, namely short distances between structures, its accurate replication imparts characteristics of hydrophobicity to the polymeric samples.

### 3.4. Determination of Relevant Sample Surface Parameters

**Figure 8:**
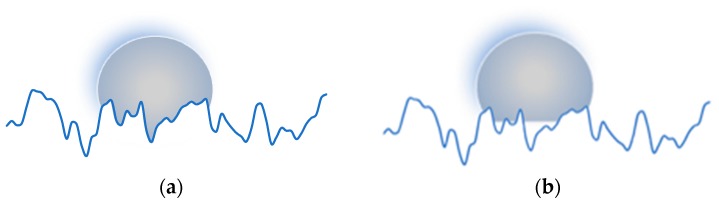
(**a**) Scheme of the Wenzel model and (**b**) Cassie–Baxter model applied to a random surface.

The differences observed in the topography of the samples obtained with different evolutions of the cavity surface temperatures are responsible for the different hydrophobicity. Two models are considered to correlate the hydrophobicity to the surface topography: Wenzel [43] and Cassie–Baxter [44]. In the Wenzel regime, the hydrophobicity of a rough surface is enhanced by the increase of the solid-liquid contact area. In the Cassie–Baxter regime, wetted surface is composed by the solid and by the air, which results to be trapped between the droplet and the solid, in this case, the hydrophobicity increased by the decrease of the solid-liquid contact area. Figure 8 schematizes the two models applied to a random surface.

Wenzel and Cassie–Baxter models are reported in the Equations (1) and (2), respectively.

(1) $$\cos\theta =r\;{\cos\theta }_{0}$$

(2) $$\cos\theta =f\cos\theta_{0}-\left(1-f\right)$$

The θ_0_ is the Young contact angle corresponding to an ideal smooth surface. In this paper, the Young contact angle was measured on moldings obtained in the conditions named CIM, in which the cavity surface had a nominal roughness of 15 ± 2 nm. The molding roughness, being of 10 ± 2 nm, can be considered smooth [45], and its contact angle is 80°, according to the smallest contact angle found in the moldings (see Figure 3).

The *r* is the ratio among the real rough surface area and the projected smooth surface, and it takes into account that the increase in contact angle is due to an increase of the wetted surface (which determines an increase of free energy) with respect to the projected one; *f* is a factor that enables taking into account that the droplet contacts a composed surface made of solid (on an area fraction *f*) and air (on an area fraction 1 − *f*). *r* and *f* can be calculated directly from the 3D topographic parameters, as suggested by Vogler et al. [46], *S*_dr_, *S*_da_ and *S*_ha_ mentioned in the method section [41,46]:

(3) $$r=\frac{S_{\mathrm{dr}}+100}{100}$$

(4) $$f=\frac{S_{\mathrm{ha}}}{S_{\mathrm{ha}}+S_{\mathrm{da}}}$$

The topographic parameters were evaluated for all the considered AFM topographic maps to relate these parameters to the measured contact angles.

**Figure 9:**
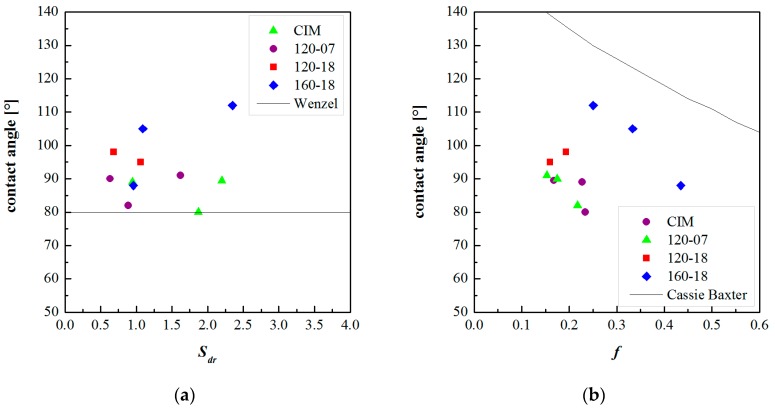
Contact angles vs. the parameters *S*_dr_ (**a**) and *f* (**b**) calculated from the AFM flattened maps. The Wenzel and the Cassie–Baxter models were also reported in each plot.

Figure 9 shows the contact angles, for the CIM sample and the samples obtained with different cavity surface temperature evolutions, as function of the parameters *S*_dr_ and *f*, respectively. The parameters *S*_dr_ and *f* were taken from the flattened AFM acquisitions. For each considered sample, the AFM acquisitions were taken in three different positions along the flow path.

**Figure 10:**
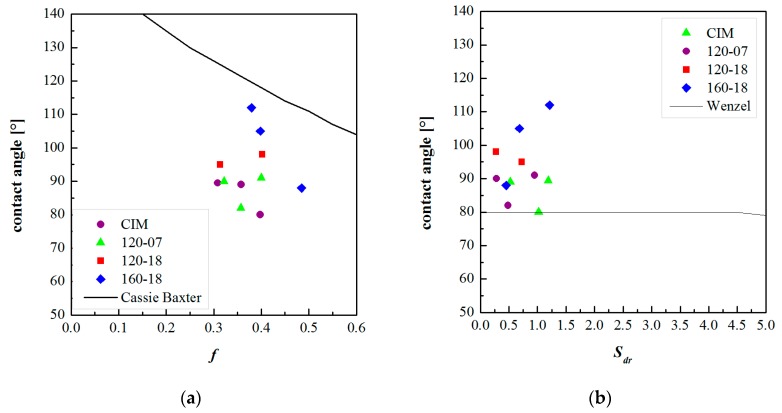
Contact angles vs. the parameters *S*_dr_ (**a**) and *f* (**b**) calculated on the AFM maps where a high pass filter of 0.2 µm^−1^ was applied. The Wenzel and the Cassie–Baxter models were also reported in each plot.

Figure 10 also shows the contact angles as function of the parameters *S*_dr_ and *f* but these parameters were calculated on the AFM acquisitions filtered with 0.2 µm^−1^ high pass cutoff frequency. Figure 9 and Figure 10 reports also the Wenzel and the Cassie–Baxter model as black lines.

Figure 9a and Figure 10a show that there is not a relationship between the contact angles and the surface parameter *S*_dr_. Thus, the Wenzel model does not fit the experimental data.

The Cassie–Baxter model does not fit the experimental data; however, this model appears to be more suitable, especially for the sample 160-18, in describing the relationship between the measured contact angles and the surface characteristics. Figure 9b and Figure 10b show that the parameter *f* has to be higher with respect to the calculated ones. The reason for this discrepancy could be due to the method adopted for calculating *f*, which does not take into account the overall complexity of the surface. The real surfaces are composed of structures on different scales, both micro and nano-metrical, with a complex harmonic content (see Figure 7). This aspect is not considered in the calculation of *f*, since *S*_da_ and *S*_ha_ are referred to projected areas; furthermore, they have averaged values that could be not representative of the real surface topography. The real surface is characterized by structures with a certain height and by different frequencies of dales and hills, this information has to be taken into account together in the calculation of the surface parameters.

## 4. Discussion

The steel layer adopted as cavity surface in this work is characterized by randomly dispersed structures (see Figure 5 and Figure 6). These structures present micro and nano-metrical heights, i.e., roughness, and spatial distances between structures down to the nano-metrical level. The roughness is obviously not a good measure of the surface hydrophobicity, since surfaces showing similar roughness present quite different contact angles. The frequency analysis of the acquired patterns provides instead a method to check the replication accuracy both in the structure height and spatial distribution (Figure 7). The replication is enhanced by the temperature and heating time [47,48].

It is necessary to operate with long heating times to reach an accurate replication both in height and in the spatial distance, since the replication of structures is due to the filling of micro and nano-cavities by a pressure-driven mechanism. The temperature has to be high during the filling of the micro or nano-cavities to prevent solidification and crystallization that inhibit an accurate replication.

The moldings that show the most accurate replication of structures also show the highest hydrophobicity. In particular, the hydrophobicity increases for the surfaces showing the replication of structures characterized by high spatial frequencies, namely nano-metrical distances between structures.

The two models considered, Wenzel and Cassie–Baxter, identify relationships between the surface topography and the hydrophobicity. The latter model appears to better describe the behavior of the contact angles with respect to the surface topography. The discrepancy between the Cassie–Baxter model and the experimental findings might be due to the method adopted to evaluate the fraction *f* of the liquid in contact with the solid rather than with trapped gas, through the topographic parameters *S*_da_ and *S*_ha_, which might be not suitable to describe a real liquid wetted surface. In particular, the trapped air prevents the complete filling of the dales by the liquid. While the topography of the surfaces proposed in the literature [26,41], being formed by isolated reliefs uniformly spaced on the surface, allows for a partial escaping of the trapped air, a random surface with closed dales could present limited escape routes for air. Furthermore, on a real surface, the liquid filled levels depend also on the pressure that the trapped air has to withstand, since its volume decreases with pressure. In addition, the topographic parameters *S*_da_ and *S*_ha_, evaluated in this work according to the standards, are not representative of the real surface topography, being simply referred to the averaged projected areas of dales and hills; thus, neglecting the effect of their heights. Following the standard and also the literature [41], all these aspects were neglected in the calculation of the topographic parameters, and thus in the calculation of *f*; this may have determined a relevant part of the discrepancy between the Cassie–Baxter model predictions and the experimental findings, as shown in Figure 9.

## 5. Conclusions

In this work, a very efficient and new technique was adopted to obtain the evolution of the temperature on the cavity surface and to tune hydrophobicity of polyolefins. The heating device adopted to achieve the fast evolution of the cavity temperature allows for tuning the temperature locally, and it is also possible to keep constant the selected temperature for the desired time. Being limited to a small thickness below the cavity surface, the cooling after the heater deactivation results to be very fast, with a rate comparable with the cooling rate of the conventional injection molding process. This technique is very efficient in replicating structures, both in the height and shape, in a wide dimensional range, micro and nano-metric. By adopting this technique, the effect of the operating conditions on the hydrophobicity of molded iPP samples was analyzed. Furthermore, the correlation among the hydrophobicity and the surface topography of the moldings was explored. The replication of a random surface, composed by both sub-micro and nano-structures, was analyzed under different injection molding conditions. The presence of sub-micro and nano-structures characterized by high values of spatial frequency was detected and highlighted only in the molded samples obtained with high cavity surface temperatures.

The static water contact angles were measured on all molded samples at different distances from the gate along the flow path. The contact angles were found to be higher in the heated area and lower in the unheated area. A significant increase of the contact angle was measured when high cavity surface temperatures and long heating times were adopted during injection molding. Small heating times, comparable with the cavity filling time, were not sufficient to induce significant increases of the contact angles.

The experimental contact angles were plotted versus the main surface parameters, calculated from AFM acquisitions, to find correlations between sample surface morphology and the increase of the contact angle with respect to the Young value. Both the Wenzel and the Cassie–Baxter models were compared with the experimental data. It was found that the behavior of the contact angle with respect to the surface parameter *f* (which estimates the fraction of surface where the liquid is in contact with the solid rather than with trapped gas) was close to the behavior described by Cassie–Baxter model, especially for the sample obtained with the highest cavity surface temperature and the longest heating time. The residual discrepancy between the theoretical model and the experimental data could be attributed to the complexity of the description of both the sample surface topography and the split of water droplet contacts between the polymer and the entrapped air, which could not be adequately described into the parameter *f*.

## Figures and Tables

**Table 1:** Operative injection molding conditions (*T*_melt_ = melt temperature; *T*_mold_ = mold temperature; *P*_holding_ = holding pressure; *T*_level_ = temperature reached on the cavity surface thanks to the activation of the heating device).

Test	*T*_melt_ [°C]	*T*_mold_ [°C]	*P*_holding_ [MPa]	Holding time [s]	*T*_level_ [°C]	Heating time [s]
CIM	220	28	26	2	28	0
80-07	220	28	26	2	80	0.7
120-07	220	28	26	2	120	0.7
160-07	220	28	26	2	160	0.7
80-18	220	28	26	2	80	18
120-18	220	28	26	2	120	18
140-18	220	28	26	2	140	18
160-18	220	28	26	2	160	18

**Table 2:** Roughness of the steel layer and of the molded samples CIM and 160-18 at 20 mm distance from the gate.

Test	Filter	5 mm	20 mm	55 mm
**Steel layer**	flattened	164 ± 30 nm		
	0.1 µm^−1^	64 ± 10 nm		
	0.2 µm^−1^	25 ± 5 nm		
**CIM**	flattened	113 ± 25 nm	139 ± 10 nm	116 ± 10 nm
	0.1 µm^−1^	48 ± 3 nm	38 ± 5 nm	48 ± 3 nm
	0.2 µm^−1^	26 ± 3 nm	20 ± 2 nm	25 ± 2 nm
**160-18**	flattened	77 ± 5 nm	136 ± 15 nm	106 ± 12 nm
	0.1 µm^−1^	25 ± 5 nm	40 ± 6 nm	24 ± 2 nm
	0.2 µm^−1^	23 ± 5 nm	27 ± 3 nm	23 ± 2 nm
